# Online and Social Networking Interventions for the Treatment of Depression in Young People: A Systematic Review

**DOI:** 10.2196/jmir.3304

**Published:** 2014-09-16

**Authors:** Simon M Rice, Joanne Goodall, Sarah E Hetrick, Alexandra G Parker, Tamsyn Gilbertson, G. Paul Amminger, Christopher G Davey, Patrick D McGorry, John Gleeson, Mario Alvarez-Jimenez

**Affiliations:** ^1^Orygen Youth Health Research CentreCentre for Youth Mental HealthUniversity of MelbourneMelbourneAustralia; ^2^headspace Centre of Excellence in Youth Mental HealthUniversity of MelbourneMelbourneAustralia; ^3^Department of Child and Adolescent PsychiatryMedical University of ViennaViennaAustria; ^4^School of PsychologyAustralian Catholic UniversityMelbourneAustralia

**Keywords:** Internet, depression, young adult, adolescent, social networking, support groups, review

## Abstract

**Background:**

Major depression accounts for the greatest burden of all diseases globally. The peak onset of depression occurs between adolescence and young adulthood, and for many individuals, depression displays a relapse-remitting and increasingly severe course. Given this, the development of cost-effective, acceptable, and population-focused interventions for depression is critical. A number of online interventions (both prevention and acute phase) have been tested in young people with promising results. As these interventions differ in content, clinician input, and modality, it is important to identify key features (or unhelpful functions) associated with treatment outcomes.

**Objective:**

A systematic review of the research literature was undertaken. The review was designed to focus on two aspects of online intervention: (1) standard approaches evaluating online intervention content in randomized controlled designs (Section 1), and (2) second-generation online interventions and services using social networking (eg, social networking sites and online support groups) in any type of research design (Section 2).

**Methods:**

Two specific literature searches were undertaken. There was no date range specified. The Section 1 search, which focused on randomized controlled trials, included only young people (12-25 years) and yielded 101 study abstracts, of which 15 met the review inclusion criteria. The Section 2 search, which included all study design types and was not restricted in terms of age, yielded 358 abstracts, of which 22 studies met the inclusion criteria. Information about the studies and their findings were extracted and tabulated for review.

**Results:**

The 15 studies identified in Section 1 described 10 trials testing eight different online interventions, all of which were based on a cognitive behavioral framework. All but one of the eight identified studies reported positive results; however, only five of the 15 studies used blinded interviewer administered outcomes with most trials using self-report data. Studies varied significantly in presentation of intervention content, treatment dose, and dropout. Only two studies included moderator or clinician input. Results for Section 2 were less consistent. None of the Section 2 studies reported controlled or randomized designs. With the exception of four studies, all included participants were younger than 25 years of age. Eight of the 16 social networking studies reported positive results for depression-related outcomes. The remaining studies were either mixed or negative. Findings for online support groups tended to be more positive; however, noteworthy risks were identified.

**Conclusions:**

Online interventions with a broad cognitive behavioral focus appear to be promising in reducing depression symptomology in young people. Further research is required into the effectiveness of online interventions delivering cognitive behavioral subcomponents, such as problem-solving therapy. Evidence for the use of social networking is less compelling, although limited by a lack of well-designed studies and social networking interventions. A range of future social networking therapeutic opportunities are highlighted.

## Introduction

Major depressive disorder is reported as the leading cause of disability worldwide [1] and accounts for the greatest burden of all diseases globally [2-4]. The effects of depression are considerable and are associated with a range of negative outcomes [5]. Depression typically first manifests during adolescence or young adulthood (up to 25 years) [6] and tends to display a worsening pattern over the course of repeated episodes, including a lack of responsiveness to initially effective treatments [7]. Relapse rates after a first episode of depression are 20-30%, compared to 70-80% for those who have experienced two or more episodes [8], and research has established that relapse rates in children and adolescents range between 34% and 75% within 1-5 years after the first depressive episode [9].

Cognitive behavioral therapy (CBT) is recommended as a first line treatment for depression [10-13]. However, research has suggested that CBT may be only modestly effective in treating depression in young people [14]. Furthermore, mental health service use is low among young people for a variety of reasons, including distrust of health professionals and stigma [15]. Hence, there is a need for better youth depression treatment strategies that maximize engagement and reduce likelihood of relapse or recurrence [16,17].

Given young people’s enthusiasm for Web-based communication, the development of innovative, online psychosocial interventions may help improve treatment acceptability and engagement for young people experiencing depressive symptoms [13]. Further, social networking sites (SNS) are rapidly becoming an essential avenue for social communication and support [18], especially among young people [19], and could be pivotal in engagement with mental health services [20]. Using such avenues of support in the treatment of depression may also have some benefit.

In recent years, a range of online interventions have been successfully tested for the management of a number of mental disorders, with research supporting the efficacy of these interventions in alleviating anxiety and depressive symptoms [21-23]. Online interventions have been reported as being as effective as face-to-face therapy [15], and some countries now recommend the use of online interventions within clinical guidelines for the treatment of depression [24].

To date, there has been a relative lack of online intervention studies focusing on young people with depression [25]. While young people tend to choose face-to-face support as their preferred mode of help-seeking for depression [26], a significant number also indicate preference for online intervention given the added anonymity and immediacy associated with the online environment [27]. Furthermore, online interventions have the potential to reach young people who may not be inclined or able to seek help from traditional sources given their ability to transcend geographical boundaries and provide 24-hour accessibility and reach [15].

Service delivery gaps in the provision of youth specific psychotherapeutic services [28], in addition to modest treatment outcomes [14], have resulted in unmet need with adverse consequences for both the affected young people and their wider communities [15]. Consequently, there has been an increase in research evaluating the plausibility of online interventions for the treatment and relapse prevention of depression. The aim of the present study was to systematically appraise the relevant literature of online approaches to depression treatment in young people. This approach was undertaken using separate search strategies to identify studies of (1) standard online interventions, and (2) second-generation online interventions using social networking functions.

## Methods

### Searching

Electronic searches were conducted in June 2013 of the PsycINFO, Cochrane Library, Embase, and MEDLINE databases accessed via the University of Melbourne library. Search strategies were devised using relevant subject headings for each database. In order to capture all relevant online interventions, additional free text words were identified by experts who have published in youth mental health online interventions and systematic review methodology. All studies retained for analysis needed to meet the study inclusion criteria outlined below. Further manual searching of reference lists was also undertaken, in addition to a “soft search” in the Google Scholar database for Section 1 for “Online interventions” and Section 2 for “Social networking and depression”. Search terms are listed in Multimedia Appendix 1. No date range was specified.

### Inclusion Criteria

#### Section 1: Online Interventions for Depression

Included studies had to meet each of the following criteria: (1) use a randomized controlled trial (RCT) design, (2) test any therapeutic modality delivered online, (3) use either an open access (not restricted) or referred (participation through invitation only and supplied with a password to access site) intervention, (4) focus on prevention (using a universal or targeted approach), treatment, or relapse prevention, and (5) include participants aged between 12 and 25 years.

#### Section 2: Social Networking and Depression

Included studies met each of the following criteria: (1) focus on social networking services, defined as online interactions where the reader is also able to write, and where the social network is predefined, (2) either examine social networking websites that aim to prevent, treat, or provide relapse prevention for depression, or describe associations between the use of general social networking sites and depressive symptoms, and (3) include user-to-user contact. Given the early development stage of social networking interventions, no restrictions were placed on participant age or study design.

### Data Synthesis

For each section, search results were independently screened by 2 researchers to establish their relevance for inclusion in the appropriate section. Any discrepancy between the researchers was referred to a third researcher involved in establishing the search strategy for the final decision. In order to assist the narrative synthesis of the included studies, a predefined data extraction template was designed for Sections 1 and 2.

## Results

### Section 1: Online Interventions for Depression

#### Study Characteristics

We identified 101 articles, of which 15 studies met inclusion criteria (see Multimedia Appendix 2). According to characteristics of samples recruited, these studies were categorized as either prevention (ie, participants identified as at risk of depression or not meeting current diagnostic criteria for major depressive disorder [MDD], n=9), intervention (ie, participants identified as meeting clinical diagnostic criteria for MDD, n=5), or combined prevention and intervention (ie, mixed sample of participants meeting and not meeting diagnostic criteria for MDD, n=1). None of the identified 15 studies focused on relapse prevention post acute-phase treatment.

With the exception of the one combined prevention and intervention study [29], all included trials reported positive findings. However, only four trials used blinded observer ratings [24,30-32]. The majority (n=9) of trials used participant self-report data as outcome variables.

The 15 identified studies reported on data from 10 separate trials. Of these, there were nine separate interventions tested: Cognitive Behavioral Analysis [33], MoodGYM [21,34], Cognitive Behavioral Skills Training Program [35], SPARX [31,36], CATCH-IT [24,30,32,37,38], Internet Problem Solving Therapy [29], Blues Blaster [39], Computerized CBT [40], and Master Your Mood [41]. There was one secondary publication for MoodGYM and four secondary publications for CATCH-IT. All of these interventions used a cognitive behavioral treatment approach, targeting either depression, or depression and comorbid anxiety. With the exception of the Cognitive Behavioral Analysis intervention [33], each of the interventions were designed to be completed in modules. The number of modules ranged from 4 (Cognitive Behavioral Skills Training Program) to 14 (CATCH-IT). Based on descriptions of the intervention modules presented in the published papers, varied key CBT content tended to be present (eg, psychoeducation, behavioral activation, thought monitoring), though there was some variation across interventions. None of the included studies incorporated ongoing social networking.

The included MoodGYM, Cognitive Behavioral Analysis, and SPARX interventions were delivered via computer in a group classroom setting, with supervision from a classroom teacher/tutor. The remaining interventions were designed to be delivered in a self-paced and self-directed manner. Studies varied in duration (eg, dose) both in terms of total length of intervention access, which ranged from 5 weeks (eg, MoodGYM) to 32 weeks (eg, Cognitive Behavioral Skills Training Program), and individual module length, which ranged from 20-40 minutes (MoodGYM) to 90 minutes (Master Your Mood).

The majority of included studies involved a fully automated intervention, with only two of the eight interventions including moderator involvement, or feedback to participants. Of the moderated interventions, the Internet Problem Solving Therapy intervention used email support from the moderator (with messages followed up within a 3-day period) [29], plus several phone calls to monitor safety. The Master Your Mood intervention adopted a closed chat-room moderation style. This was facilitated by trained professionals, and the intervention content was presented in an online group format (6 sessions, 90 minutes duration, 6 participants maximum) [41]. The CATCH-IT intervention included several motivational phone calls, provided only to those in the motivational interviewing condition.

Dropout rates were reported for 14 of the 15 studies and varied significantly, ranging from 3%-41%. The only study with a dropout rate of less than 5% used the SPARX avatar-based gaming intervention platform [36] with the accompanying RCT of SPARX [31] also reporting a low dropout rate (9.6%). Four studies [29,35,39,41] reported attrition rates above 15%. These studies varied in their level of moderator and peer contact: two were fully automated (Cognitive Behavioral Skills Training; Blues Blaster) [35,39], one used occasional moderator phone contact (Internet Problem Solving Therapy) [29], and one included direct peer-to-peer contact via chat room sessions (Master Your Mood) [41].

#### Efficacy: Prevention Studies

The prevention studies (Blues Blaster, Cognitive Behavioral Analysis, MoodGYM, and CATCH-IT) demonstrated intervention efficacy among university students [33,39], secondary students (although effects were significant only for male participants) [21], and adolescents identified as at-risk of depressive disorder [24,37,38,42,43]. Several studies using the CATCH-IT intervention indicated that motivational interviewing provided by a primary care practitioner prior to intervention commencement aided the young person’s engagement and symptom improvement [24,37,38]. However, the CATCH-IT intervention had not been compared to no intervention or a comparison intervention. For the prevention studies, comparison groups varied from waitlist control, attention placebo, and those not receiving motivational interviewing.

#### Efficacy: Intervention Studies

The intervention studies demonstrated superiority of the online intervention to the comparison treatment. These involved the Cognitive Behavioral Skills Training Program, SPARX, Computerized CBT, and Master Your Mood. Comparison groups were either treatment as usual (pharmacotherapy and psychosocial services) [35], waitlist control [36,41], or brief psychoeducation [40]. One of the more novel intervention studies (the large SPARX RCT intervention that integrated CBT concepts within an online fantasy game) demonstrated non-inferiority relative to treatment as usual provided by youth clinics, school-based counseling services, or general practitioners [31].

#### Combined Prevention and Intervention

The trial that targeted its Internet Problem Solving Therapy intervention to young people who were either at risk of depression or had depression showed no difference between the intervention group and the waitlist control. This trial recruited a relatively small (n=45) yet heterogeneous sample [29]. Participants were 12-18 years, with mild/moderate depressive and/or anxiety symptoms. Depressive and anxiety symptoms declined in both the intervention and waitlist control groups, with no added benefit of the Internet-delivered problem solving intervention.

### Section 2: Social Networking and Depression

#### Study Characteristics

A total of 358 abstracts were screened, yielding 22 studies meeting inclusion criteria (see Multimedia Appendix 2). Articles were grouped into three overarching categories: (1) social networking sites (SNS, n=16) [44-59] defined as sites with a non-specific user purpose, (2) online support groups (OSG; n=4) [22,60-62] defined as sites with a specific purpose of seeking and sharing health-related information and related personal experiences, and (3) general commentary (n=1) [63]. One additional study undertook a combined analysis of SNS and OSG use [64]. There were 8 studies with a clinical focus (eg, recruiting help-seeking individuals) and 14 studies recruiting from a general population. None of the SNS or OSG studies focused on relapse prevention post acute-phase treatment.

The majority of included studies reported on data collected via online questionnaires. All but five of the identified SNS studies reported on participant data, with the remaining studies including one systematic review, one general literature review, two pieces discussing SNS in the context of clinical practice, and a descriptive networking study. Ages ranged between 12-85 years; however, with the exception of the systematic review paper [22], the two case study reports [54,59], and the analysis of online support group postings [60], all included studies focused on young people (eg, those aged 25 years or younger).

#### Studies Focusing on Social Networking Sites

Findings related to the benefits of SNS on depression were inconsistent. Eight of the SNS studies reported positive findings [46,48-50,55,56,58,59], five reported mixed or unclear findings [45,47,53,54,64], and three studies reported negative findings [44,51,52]. The SNS studies are discussed relative to potential benefits and harms.

#### Potential Benefits of Using Social Networking Sites

Direct one-to-one interactive communication (as opposed to passive SNS use) was associated with greatest well-being and least depression [45]. SNS use was associated with potential mental health benefits including socialization, facilitation of supportive relationships, belongingness, self-esteem, communication, and learning [46,63]. Positive findings highlighted key opportunities in the relationship between SNS use and depression for those from marginalized groups at risk of social isolation [46]. This study also highlighted the ubiquitous nature of young people’s use of SNS and benefits of educational outcomes, facilitating supportive relationships, identity formation, and self-esteem. Three studies using an online questionnaire design identified the potential for SNS to aid in the prevention of symptom onset and symptom remission by functioning as a screening tool for at-risk individuals via status update posts [49,50,55]. Potential interventions, such as targeted advertising triggered by keywords posted on social networking sites, could present relevant cost-effective options for mental health support [55]. Social networking was also identified as a means to re-establish friendships following social withdrawal for those experiencing depression [59].

#### Potential Harms of Using Social Networking Sites

Identified risks included cyberbullying, harassment, sexting, privacy concerns, and SNS-induced depression in the association between young people’s use of SNS and depression [63]. As expected, negative interactions with SNS were associated with greater depressive symptomology and negative affect [44,57]. However, at the general population level, there was little evidence of a direct relationship between depression and general SNS use [47,48]. This was highlighted by a weak relationship (*r*=.15) between high school students’ use of SNS and depression symptoms [51]. Although use of SNS was identified as both a possible causal contagion factor in a cluster of suicides among 15-18 year olds [52], suicidal posts on SNS were also identified as offering an opportunity for identification and intervention [54].

#### Studies Focusing on Online Support Groups

The included systematic review of OSG use reported a lack of high-quality research examining the effects of OSGs on depression. The included systematic review reported that only two low-quality studies examined the effectiveness or efficacy of OSG use on depression with mixed results [22]. This finding is echoed in the present review, with no RCTs found that test OSG interventions for depression.

Of the included OSG studies in our review, three reported overall positive findings [60-62] and recommended OSGs for people experiencing depression. These studies reported that regular participation in OSGs was associated with participants receiving greater online and subsequent offline emotional support for depression and that OSG use may complement formal care, provide an environment for knowledge exchange, and inspiration for coping with depression [60].

In contrast, one study reported that use of OSGs (but not SNS) was associated with increased suicidal ideation for young people 14-24 years over a 12-month period [64]. Other general disadvantages of OSG membership included reports that some individuals experienced participation as burdensome (and experienced symptom exacerbation) following negative comments posted by other site members [57]. Further, excessive reliance on online support was also apparent for some individuals. Preference for online support tended to be evident in cases where external supportive networks were not apparent [65].

## Discussion

### Principal Findings

Young people are eager users of new technology, and online interventions offer an opportunity for mental health support that is immediate and cost-effective [15]. This review draws together two threads of emerging inquiry related to young people’s use of online technology for managing symptoms of depression. Findings related to RCTs of online interventions in young people indicated largely positive effects. This is consistent with broader findings from reviews of Web-based interventions undertaken with adult populations [66] and university students [67,68]. The overall impact of SNS use was less clear but may in part be due to significant heterogeneity in research design, target population, and intervention assessed.

### Online Interventions and Depression

With the exception of Internet Problem Solving Therapy, all studies highlighted the benefits of online interventions for prevention and treatment of major depression in young people. However, caution is required in interpreting this finding given that few studies used blinded interviewer outcome assessments. General recommendations included the feasibility of widespread roll-out of online interventions across school-based populations [21], comparative low cost [35], applicability to group-based delivery [41], appeal to young people outside of mainstream educational settings [36], and the ability of online interventions to assist with unmet clinical need [31]. Furthermore, 6-month [30] and 12-month [32] follow-up of the CATCH-IT intervention suggests enduring clinical benefits. However, the CATCH-IT intervention has not been evaluated against a competing intervention or control group.

Based on sample characteristics, studies were classified as prevention, intervention, or both. However, in practice, included studies tended to make little distinction regarding this and were broad in their disorder of focus. For example, the MoodGYM intervention is designed to both prevent and decrease depression symptoms [21], the Blues Blaster intervention was evaluated as a prevention intervention, despite being adapted from the Adolescent Coping With Depression clinical intervention [39,69], and the Internet Problem Solving Therapy intervention was designed to treat both depression and anxiety [29]. A logical advance for the field would be greater specificity in the development and testing of online interventions that specially match treatment focus to stage of disorder (eg, primary prevention, clinical intervention, relapse prevention).

Participant attrition is an important issue in the use of online interventions. Included studies that promoted greater engagement (either through use of motivational interviewing or content innovation) tended to report lower attrition rates. Automated self-help services require significant motivation and self-discipline [29], and this may be expecting too much from young people experiencing depression. Indeed, low motivation of young people and possible dissatisfaction with delayed moderator/clinician input were offered as an explanation for outcomes of the only included Section 1 study to report no benefit of the online intervention [29].

Ongoing engagement is also an important factor for online interventions. The effect of high intervention adherence (relative to low intervention adherence) was found to improve treatment outcomes of the MoodGYM intervention [34]. This is a noteworthy finding given some interventions report relatively low levels of engagement (eg, the CATCH-IT study reported that 9% and 22% of those allocated to respective treatment groups failed to access the intervention at all [24]).

### Social Networking—Therapeutic Opportunities

The primary focus of the studies reviewed in Section 2 was the exploration of the relationship between engagement in SNS (including OSG use) and depressive symptomology. This is an emerging research area, and there was significant heterogeneity across study designs. Hence, results must be interpreted with caution.

Research suggests that some young people may be more willing to disclose information on an SNS than in person [70]. While SNS use may result in unhealthy online interactions for some [63], for many vulnerable young people SNSs may provide needed opportunities for social support and identity formation [45]. Indeed, longitudinal research has shown that Internet use for social support is associated with declines in depression [71]. Because of the noted risks (alongside identified benefits) of SNS use for depression, researchers and clinicians must work towards the development of protocols to integrate such functionality within a safe and supportive framework and provide positive alternatives to non-therapeutic SNS.

Given the early phase of mental health–related research on the use of SNS, significant knowledge gaps in research are to be expected. While some of the SNS studies reported positive findings, others identified adverse outcomes or no associations at all. It is possible that SNSs exert a bidirectional effect on depression symptoms or that the relationship is mediated by the intervention content, safety, and type of interaction. For example, positive online interactions may lead to increased social support and reduced depression, and negative online interactions (or with a negative focus) may lead to increased depression and perceived burden.

There may also be qualitative differences between young people who use particular websites for help seeking or discussion of mental health issues. For example, previous research has shown that those higher in hopelessness (a key predictor of suicidal ideation) may be more likely to engage in blogging type sites (eg, OSGs) versus sites focused on briefer posts and content [64]. OSGs are communities designed to provide support, acceptance, and knowledge exchange between members overcoming similar adversities and may attract young people with particular personality characteristics. Given the overall study design limitations (eg, lack of control or comparison group), well-designed trials are urgently needed in this area [22].

Participation in online social networking has become ubiquitous and routine in the lives of young people. As such, there is both a great opportunity and an urgent need to identify and make use of the potential benefits of such sites for young people’s well-being. Provided that the SNS experience is primarily positive, time spent on SNSs has the potential to operate as a preventative or therapeutic medium for individuals with depression and may complement traditional therapy for more severe forms of the disorder.

### Implications and Limitations

Adherence and engagement are essential to effective and efficient delivery of online interventions [34]. Although the population reach and cost-effectiveness of online interventions makes their use attractive, significant work remains to be done in refining and better targeting online interventions to maximize their effectiveness. As online interventions evolve, they will incorporate greater dynamism and functionality. It is possible that automated self-help interventions may lose their appeal, and the development of future interventions must consider features and treatment strategies that promote engagement. A further and necessary advancement, given potential risk issues associated with unmonitored suicidality or symptom deterioration, will likely include the use of real-time moderator input and integrated crisis support within the online environment. This was lacking in the studies identified in our review. Emerging models of online moderation are evolving, including improving the integration of clinician input into online interventions [25]. For example, the moderated online social therapy (MOST) framework [72-74] provides a methodology for promoting ongoing engagement through online therapy, social networking, and regular clinician support for relapse prevention of serious mental health problems in young people.

The next generation of online interventions will include refinements and functionality not possible in previous technologies. There is significant scope for greater responsiveness and immediacy, including real-time clinician input and customized feedback. In addition, the next generation of online interventions offers an opportunity for better matching of intervention content to phase of illness, and the role of online social and peer support [26,72].

Literature searching for the present review was current at June 2013, and it is likely that higher-quality studies evaluating SNS use will start to soon appear in scholarly journals. Promising new lines of research are emerging for the treatment of depression in young people using mobile phone-based interventions [75,76], live chat interventions [77], Web-based positive psychology interventions [78], and interventions focusing on comorbid depression and alcohol misuse [79]. Importantly, the current review did not include studies reporting on data from Twitter. Recent work using sentiment analysis and qualitative methodologies has highlighted that relative to those without depressive symptoms, Twitter users who experience depressive symptoms are more likely to post references to negative emotions or anger [80] and are more likely to view Twitter as a tool for emotional interaction and consoling oneself [81]. Complementing this work, emerging evidence suggests that salient emotional content from Twitter posts may effectively predict depression before actual symptom onset [82]. Taken together, these findings suggest potential broad implications for scalable early detection and intervention, and they highlight the need for a comprehensive review of studies using Twitter generated data.

The present review was limited by a lack of studies focusing on relapse prevention. Given that relapses in depression are high [7,8] the lack of relapse prevention studies using SNS or OSGs is concerning and is an area requiring urgent research and clinical attention. Illustrating this, a recent Cochrane review highlighted the need for rigorous RCTs to assess intervention efficacy and psychotherapy interventions aimed at preventing relapse given the lack of progress in this area [3]. Further, all included studies in Section 1 focused on cognitive behavioral interventions. Given that research suggests that CBT may be only modestly effective with young people [14], there is value in developing and evaluating relapse prevention interventions that draw on other therapeutic modalities (eg, strengths-based approaches) [83]. Hence, researchers and clinicians should focus their energies on the development and evaluation of innovative online treatments for depression relapse prevention.

### Conclusions

Mental health interventions will increasingly make use of online technologies. A key challenge for these interventions will be having broad appeal and engagement. There is clear evidence that online interventions using a cognitive behavioral focus are promising in reducing depression symptomology in young people. Further study is required to identify the effectiveness of online interventions delivering cognitive behavioral subcomponents, such as problem solving therapy or other therapeutic approaches. Although evidence for the use of online social networking is less robust, such interventions exist in a dynamic space with significant opportunity for methodological advancement and rigor.

## Multimedia Appendix 1

Search terms.

## Multimedia Appendix 2

Summary of studies, Sections 1 and 2.

## Abbreviations

CBT: cognitive behavioral therapy
MDD: major depressive disorder
OSG: online support group
RCT: randomized controlled trial
SNS: social networking site
