# Supplementary material for: Online and Social Networking Interventions for the Treatment of Depression in Young People: A Systematic Review
Source: J Med Internet Res. 2014 Sep 16;16(9):e206. doi: 10.2196/jmir.3304 (PMC4180352; doi:10.2196/jmir.3304)
Supplement: Supplementary file 1 [file jmir_v16i9e206_app1.pdf]

Multimedia Appendix 1.

Section 1 search terms

Search	Terms
S1	((MM "Depression (Emotion)") OR (MM "Major Depression")) or (TI depressive disorder* or AB depressive disorder* or TC depressive disorder* or KW depressive disorder*) or ((MM "Affective Disorders") OR (MM "Dysthymic Disorder"))
S2	DE "Clinical Trials"
S3	TI controlled trial* or AB controlled trial* or TC controlled trial* or KW controlled trial*
S4	TI (controlled studies or controlled study) or AB (controlled studies or controlled study) or TC (controlled studies or controlled study) or KW (controlled studies or controlled study)
S5	TI random* or AB random* or TC random* or KW random*
S6	DE "Random Sampling"
S7	TI singl* N5 blind* or TI singl* N5 dummy or TI singl* N5 mask* or TI doubl* N5 blind* or TI doubl* N5 dummy or TI doubl* N5 mask* or TI trebl* N5 blind* or TI trebl* N5 dummy or TI trebl* N5 mask* or TI tripl* N5 blind* or TI tripl* N5 dummy or TI tripl* N5 mask*
S8	AB singl* N5 blind* or AB singl* N5 dummy or AB singl* N5 mask* or AB doubl* N5 blind* or AB doubl* N5 dummy or AB doubl* N5 mask* or AB trebl* N5 blind* or AB trebl* N5 dummy or AB trebl* N5 mask* or AB tripl* N5 blind* or AB tripl* N5 dummy or AB tripl* N5 mask*
S9	TC singl* N5 blind* or TC singl* N5 dummy or TC singl* N5 mask* or TC doubl* N5 blind* or TC doubl* N5 dummy or TC doubl* N5 mask* or TC trebl* N5 blind* or TC trebl* N5 dummy or TC trebl* N5 mask* or TC tripl* N5 blind* or TC tripl* N5 dummy or TC tripl* N5 mask*
S10	KW singl* N5 blind* or KW singl* N5 dummy or KW singl* N5 mask* or KW doubl* N5 blind* or KW doubl* N5 dummy or KW doubl* N5 mask* or KW trebl* N5 blind* or KW trebl* N5 dummy or KW trebl* N5 mask* or KW tripl* N5 blind* or KW tripl* N5 dummy or KW tripl* N5 mask*
S11	S7 or S8 or S9 or S10
S12	TI placebo* or AB placebo* or MJ placebo* or TC placebo* or KW placebo*
S13	DE Meta Analysis
S14	TI ((meta-analy* or metaanaly* or meta analy*)) or AB ((meta-analy* or metaanaly* or meta analy*)) or TC ((meta-analy* or metaanaly* or meta analy*)) or KW ((meta-analy* or metaanaly* or meta analy*))
S15	TI systematic review* or AB systematic review* or TC systematic review* or KW systematic review*

S16	DE "Treatment Guidelines"
S17	TI guideline* or AB guideline or TC guideline or KW guideline
S18	TI consensus or AB consensus or TC consensus or KW consensus
S19	DE "Treatment Effectiveness Evaluation"
S20	DE "Mental Health Program Evaluation"
S21	S2 or S3 or S4 or S5 or S6 or S7 or S8 or S9 or S10 or S11 or S12 or S13 or S14 or S15 or S16 or S17 or S18 or S19 or S20
S22	de OR 'adolescence'/de OR 'adolescent'/de OR 'juvenile'/de
S23	AB juvenile* or TI juvenile* or TI underage* or AB teen* or TI teen* or AB youth* or TI youth* or AB adolescen* or TI adolescen*
S24	AB 'young men' or TI 'young men' or AB 'young women' or TI 'young women' or AB 'young people' or TI 'young people'
S25	AB undergraduate* or TI undergraduate or AB college or TI college or AB student* or TI student* or AB "high school" or TI "high school" or AB "young adults" or TI "young adults"
S26	S22 or S23 or S24 or S25
S27	AB online or TI online or KW online or AB computer or TI computer or KW computer or AB web or TI web or KW web or AB internet or TI internet or KW internet
S28	AB "relapse prevent*" or TI "relapse prevent*" or KW "relpase prevent*" or AB prevent or TI prevent* or KW prevent*
S29	S1 and S11 and S21 and S26 and S27 and S28
S30	S1 and S26 and S27 and S28
S31	S1 and S26 and S27 and S28

Section 2 search terms

Search ID	Search Terms
#1	"Facebook" or "myspace" or "social network*" or "social media":ti,ab,kw
#2	MeSH descriptor: [Computer Communication Networks] explode all trees
#3	MeSH descriptor: [Social Networking] explode all trees
#4	#1 or #2 or #3
#5	MeSH descriptor: [Depressive Disorder] explode all trees
#6	MeSH descriptor: [Suicide, Attempted] explode all

	trees
#7	MeSH descriptor: [Self Mutilation] explode all trees
#8	MeSH descriptor: [Suicide, Attempted] explode all trees
#9	MeSH descriptor: [Suicidal Ideation] explode all trees
#10	#5 or #6 or #7 or #8 or #9
#11	#4 and #10
