# Supplementary material for: Online and Social Networking Interventions for the Treatment of Depression in Young People: A Systematic Review
Source: J Med Internet Res. 2014 Sep 16;16(9):e206. doi: 10.2196/jmir.3304 (PMC4180352; doi:10.2196/jmir.3304)
Supplement: Supplementary file 2 [file jmir_v16i9e206_app2.pdf]

Multimedia Appendix 2.

Table 1. Summary of included studies: Online interventions for depression (Section 1).

**Study, *n***	**Primary focus (design)**	**Intervention & duration**	**System moderation, peer-to-peer contact**	**Target group, dropout (pre-post), blinded interview**	**Outcomes, overall finding**
Braithwaite & Finchim (2007) [33]<br>*n*=91	Prevention: Depression, anxiety, relationship distress<br>(RCT with 3 groups)	1. CBT (depression and anxiety computer-based preventative intervention); versus<br>2. Relationship therapy (computer-based relationship focussed preventative intervention); versus<br>3. Psychoeducation (attention placebo).	No moderation, no peer-to-peer contact.	Undergrad psychology students in a romantic relationship ≥4 months.<br>Dropout rate not reported (8-week intervention)<br>No blinding, outcomes assessed by self-report.	Relative to control, both interventions were associated with reduced symptoms of depression and anxiety, and improvement in relationship functioning (no sig difference between the interventions). Overall positive.

		Weekly sessions for 7-weeks, session length not indicated, not modularised, not interactive, delivered in university tutorials.			
Calear et al (2009) [21]<br>*n*=1,477	Prevention: Depression, anxiety (Cluster RCT)	1. MoodGYM (CBT-based online intervention); versus<br>2. Wait list control.<br><br>Weekly sessions for 5-weeks, ranging 20-40 mins in length, includes 5 interactive modules, delivered in school computer lab.	No moderation, no peer-to-peer contact.	Students 12-17 yrs.<br><br>Dropout of 8.7% between pre- and post-intervention assessments (5-week intervention).<br><br>No blinding, outcomes assessed by self-report.	Intervention group had lower anxiety than did waitlist controls at post intervention and 6-month follow-up; weaker effects for depression (small effect found for males at post intervention and 6-month follow up). Overall positive.
Calear et al (2013) [34]<br>*n*=1,477	Prevention: Depression, anxiety (Cluster,	1. MoodGYM (high adherers); versus<br>2. MoodGYM (low adherers); versus	No moderation, no peer-to-peer contact.	Students 12-17 yrs.<br><br>Dropout as per	No difference between high and low adherence groups. In comparison to control, high adherence

	stratified, RCT)	3. Wait list control.<br><br>As reported above in Calear et al (2009).		Calear et al (2009).<br><br>No blinding, outcomes assessed by self-report.	group reported stronger intervention effects (at post intervention and 6-m f/u) than low adherence. Overall positive.
Clarke et al (2009) [35]<br><br>*n*=160	Intervention: Depression (RCT)	1. Cognitive Behavioral Skills Training Program (online intervention); versus<br>2. TAU<br><br>4 modules, self-directed, interactive, CBT based.	No moderation, no peer-to-peer contact.	18-24yrs.<br><br>Dropout of 36.1% between pre- and post-intervention assessments (32-week intervention).<br><br>No blinding, outcomes assessed by self-report.	Small but significant intervention effect (this effect was moderate for females). Overall positive.
Fleming et al (2012) [36]<br><br>*n*=49	Intervention:<br><br>Depression (RCT)	1. SPARX (immediate intervention); versus<br>2. SPARX (delayed	No moderation, but participants received weekly phone call or in-person visit.	13-16yrs, excluded from mainstream education.<br><br>Dropout of 3%	Those in the immediate intervention reported improved outcomes (depression and remission) compared to delayed intervention

		intervention)<br><br>7 modules, completed over 5 weeks (1-2 modules/ week), each module requires 30 mins, interactive, delivered in school computer lab, CBT based.	No peer-to-peer contact.	between pre- and post-intervention assessments (5-week intervention).<br><br>No blinding, outcomes assessed by self-report.	group. Overall positive.
Hoek et al (2011) [30]<br><br>*n*=84	Prevention: Depression (RCT relative to YP's engagement with the online intervention)	1. CATCH-IT (online intervention plus motivational interviewing); versus<br>2. CATCH-IT (online intervention plus brief advice)<br><br>Initial engagement by primary care physician, no prescribed session	No, but MI group received 3 x motivational calls, and all participants received safety calls if required. No peer-to-peer contact.	Adolescents, at risk of depressive disorder, M = 17.47, SD = 2.04.<br><br>Dropout as per VanVoorhes et al (2008).<br><br>Yes, blinded phone assessment used.	Both groups reported decline in depression symptoms, sustained at 6-months. MI group demonstrated significantly fewer depressive episodes & less hopelessness at 6-months. Higher ratings of ease of use of online intervention predicted lower depressive symptom levels for all participants. Overall positive.

		length, 14 modules, self-directed, CBT / IPT based (details from VanVoorhees et al, 2009).			
Hoek et al (2012) [29]<br><br>*n*=45	Prevention & intervention: Depression and anxiety (RCT)	1. Internet Problem Solving Therapy (PST; guided self-help); versus<br>2. Wait list control<br><br>5 modules, completed one per week, clinician feedback provided to participants, self-directed, CBT based.	Yes, but was immediate. Support offered via 3 x safety phone calls. . Responses to emails actioned within 3 days. No peer-to-peer contact.	12-18 yrs, (M= 16.07), mild to moderate depressive and/or anxiety symptoms<br><br>Dropout of 41% between pre- and post-intervention assessments (5-week intervention).<br><br>No blinding, outcomes assessed by self-report.	Depressive and anxiety symptoms declined in both groups. Overall negative.
Makarushka (2012) [39]	Prevention: Depression (RCT)	1. Blues Blaster online intervention	No moderation, no peer-to-peer	11-15yrs, sub threshold	Greater improvement for the Blues Blaster condition in depression

n=161		(adapted from the Adolescent Coping With Depression course; Lewinsohn, Clarke, Hops, & Andrews, 1990); versus<br>2. Psychoeducation website<br><br>6 modules delivered 1 per week in 30 minute lessons, CBT based.	contact.	depression.<br><br>Dropout of 19.8% between pre- and post-intervention assessments (6-week intervention).<br><br>No blinding, outcomes assessed by self-report.	levels, negative thoughts, behavioral activation, knowledge, self-efficacy, and school functioning compared to the information-only control condition. Overall positive.
Merry et al (2012) [31]<br><br>*n*=187	Intervention: Depression (RCT)	1. SPARX online intervention; as per Fleming et al (2012) above; versus<br>2. TAU (face-to-face counselling)	No moderation, no peer-to-peer contact.	12-19yrs, help-seeking for depression.<br><br>Dropout of 9.6% between pre- and post-intervention assessments (8-week	Online intervention equally effective as TAU on depression outcome measure, online intervention associated with higher remission rate than TAU. Overall positive.

		Self-directed, CBT based.		intervention).<br><br>Yes, blinded interviewer used.	
Saulsberry et al (2013) [32]<br><br>*n*=58	Prevention: Depression (RCT)	1. CATCH-IT (online intervention plus motivational interviewing); versus<br>2. CATCH-IT (online intervention plus brief advice)<br><br>As per Hoek et al (2011) above, self-directed, CBT based.	No, but MI group received 3 x motivational calls, and all participants received safety calls if required. No peer-to-peer contact.	Adolescents, (M = 17.47, SD = 2.04).<br><br>Dropout as per VanVoorhes et al (2008).<br><br>Yes, blinded phone assessment used.	Significant within-group decreases in depressed mood, loneliness, and self-harm ideation in both groups. No between-group differences in depressed mood or depressive disorder at 1-year; however, fewer participants in MI group experienced a depressive episode. Overall positive.
Stasiak et al (2012) [40]	Intervention: Depression (RCT)	1. Computerized CBT (cCBT; CD ROM); versus	No moderation, no peer-to-peer contact.	13-18yrs.<br><br>Dropout of 5.9%	Online intervention group showed greater symptom improvement compared to psycho-ed group. Overall

n=34		2. Brief computerized psycho-education<br><br>7 modules, 25-35 minutes each, completed over 4-10 weeks, completed on computer within school counseling department, CBT based.		between pre- and post-intervention assessments (up to 10-week intervention).<br><br>Blinding unclear.	positive.
Van der Zanden et al (2012) [41]<br><br>*n*=244	Intervention: Depression, anxiety, sense of control (RCT)	1. Master Your Mood (MYM; online intervention); versus<br>2. Wait list control<br><br>5 sessions, each session 90 minutes duration, interactive chat-room style (max 6 per group), CBT based.	Yes, secured chat room sessions were moderated by one or two trained professionals. Yes peer-to-peer contact.	16-25yrs experiencing depression symptoms.<br><br>Dropout of 21% between pre- and post-intervention assessments (12-week intervention).<br><br>No blinding,	Online intervention groups showed significantly greater improvement in depressive and anxious symptoms, and mastery, at 3 months than the control group. Overall positive.

				outcomes assessed by self-report.	
Van Voorhees et al (2008) [37]<br>*n*=84	Prevention: Depression (RCT relative to YP's engagement with the online intervention)	1. CATCH-IT (online intervention plus motivational interviewing); versus<br>2. CATCH-IT (online intervention plus brief advice)<br><br>As per Hoek et al (2011) above, self-directed, CBT based.	No, but MI group received 3 x motivational calls, and all participants received safety calls if required. No peer-to-peer contact.	14-21 years and experienced persistent sub-threshold depression.<br><br>Dropout of 8.4% for total sample between pre- and post-intervention assessments (intervention length not stated; follow-up assessment occurred 4-8 weeks post enrolment).<br><br>No blinding, outcomes assessed by self-report.	Both groups demonstrated decline in depressed mood and increased social support by peers and reductions in depression-related impairment in school. Overall positive.
Van	Prevention:	1. CATCH-IT	No, but MI group	14-21 years and	Both groups actively used

Voorhees et al (2009a) [38]<br>*n*=83	Depression (RCT relative to YP's engagement with the online intervention)	(online intervention plus motivational interviewing); versus<br>2. CATCH-IT (online intervention plus brief advice)<br>As per Hoek et al (2011) above, self-directed, CBT based.	received 3 x motivational calls, and all participants received safety calls if required. No peer-to-peer contact.	experienced persistent sub-threshold depression.<br>Dropout as per VanVoorhes et al (2008).<br>No blinding, outcomes assessed by self-report.	the site. MI group reported greater satisfaction with the Internet based component. Overall positive.
Van Voorhees et al (2009b) [24]<br>*n*=84	Prevention: Depression (RCT relative to YP's engagement with the online intervention)	1. CATCH-IT (online intervention plus motivational interviewing); versus<br>2. CATCH-IT (online intervention plus brief advice)	No, but MI group received 3 x motivational calls, and all participants received safety calls if required. No peer-to-peer contact.	14-21 years and experienced persistent sub-threshold depression.<br>Dropout as per VanVoorhes et al (2008).	Both groups substantially engaged with website and reported declining depression scores. MI group reported decline in self-harm thoughts and hopelessness, and significantly less likely than Brief advice group to experience a depressive

		As per Hoek et al (2011) above, self-directed, CBT based.		Yes, blinded phone assessment used.	episode, or report hopelessness. Overall positive.

Table 2. Summary of included studies: Social networking and depression (Section 2).

**Authors & sample size**	**Study focus target population (general vs clinical)**	**Study design, target age group**	**Outcomes, overall finding.**	**Moderator**
Burke (2011) [45] *n*=11,701	SNS, general.	Three wave panel survey, 18-85 years.	Directed, semi-public, one-to-one communication (eg, sender composing original content) was linked with the greatest wellbeing and less depression. Passive consumption (eg reading news feeds) had no additional impact on wellbeing. Overall finding mixed.	No.
Collin et al (2010) [46] *n*=NA	SNS, general.	Literature review, 12 -29 years.	SNS use associated with significantly better education outcomes, facilitation of supportive relationships, identity formation, belonging, self-esteem. Overall finding positive.	NA.
Davila et al (2012) [47]	SNS, general.	Cross sectional survey study, 18-46 years.	SNS use not depressogenic in general for young people. However, those reporting greater negative SNS interactions also reported greater depressive	NA.

Study 1 *n*=384<br>Study 2 *n*=334			symptoms. Overall finding mixed.	
Dunlop et al (2011) [64]<br>*n*=719	SNS & OSG, general.	Qualitative Interviews, 14-24 years.	SNS not linked to increased ideation. However, Online discussion forums were. Overall finding mixed.	NA.
Feinstein et al (2012) [44]<br>*n*=301	SNS, general.	Survey, 18-34 years.	Greater depressive symptoms associated with more problematic SNS interactions and experiencing more negative affect following these interactions. Overall finding negative.	NA.
Griffiths et al (2009) [22]<br>*n*=NA	OSG, clinical.	Systematic review, median age 26-65 years.	10/16 trials reported positive effect of OSG on depressive symptoms. Overall finding mixed.	NA.
Holleran (2010) [55]<br>Study 1 *n*=204<br>Study 2 *n*=126	SNS, general.	Descriptive study, 15-58 years.	Social networking sites can be cost effective and clinically relevant tool to detecting depression. Overall finding positive.	NA.

Study 3<br>*n*=124				
Jelenchick et al (2013) [48]<br>*n*=190	SNS, general.	Survey, 18-23 years.	No relationship between SNS use and clinical depression. Overall finding positive.	NA.
Moreno et al (2012) [49]<br>*n*=224	SNS, general.	Survey, 18-20 years.	Facebook users who displayed references to depression on their profile page reported higher depression scores on PHQ-9. Peers may provide supportive comments and SNSs may increase student help-seeking behavior. Overall finding positive.	NA.
Moreno et al (2011) [50]<br>*n*=200	SNS, general.	Descriptive study, 19+ years (unreported).	25% of statuses displayed depressive symptoms and 2.5% reflected a possible MDE. Users were more likely to display depressive symptoms if contacts responded to their post, and if they used Facebook more often. SNS could be an effective means to identifying students at risk of depression. Overall finding positive.	NA.
Nimrod (2013) [61]<br>*n*=631	OSG, clinical.	Cross sectional study, 20-50 years.	Heavy users received more emotional support online and more offline improvement in support for their depression than Medium users. Medium uses reported these benefits more than light users. Overall finding positive. .	Yes, but only by website admin staff, not by researcher.
Nimrod (2013) [62]	OSG, clinical.	Survey, 12-71 years.	Four member groups were identified. 'Interested in all topics' group reported more online support for depression and offline improvement than 'Information	Yes, but only by website admin staff, not by

n=763			seekers' and 'Relatively less involved'; and more offline improvement in depressive symptoms than the 'Concerned about daily living' group. Overall finding positive.	researcher.
Nimrod (2012) [60]<br>*n*=NA	OSG, clinical.	Descriptive study, age range unreported.	Online depression communities may complement formal care and serve as a platform for knowledge exchange. Inspired and empowered participants. Overall finding positive.	Yes, but only by website admin staff, not by researcher.
O'Keefee & Clarke-Pearson (2011) [63]<br>*n*=NA	General comment, general.	Short report, < 18 years.	Social media sites include a range of potential benefits (eg, socialization, communication, learning and access to information), but also many risks e.g "Facebook depression" and Cyberbullying. Overall finding mixed.	NA.
Pantic et al (2012) [51]<br>*n*=160	SNS, general.	Survey and qualitative interviews, mean age 18 years.	Significant positive correlation between BDI-II score and time spent social networking. Overall finding negative.	NA.
Robertson et al (2012) [52]<br>*n*=NA	SNS, clinical.	Descriptive study, 15-18 years.	Suicide cluster linked through SNS. Overall finding negative.	NA.
Rogers et al (2009) [53]	SNS, general.	Comparative descriptive study,	Majority preferred face to face therapy. Those in face to face therapy disclosed more emotions than those in	No.

n=328		21-30 years.	Internet therapy. Overall finding mixed.	
Ruder et al (2011) [54]<br>*n*=1	SNS, clinical.	Case study, 28 years.	Suicide notes on SNS may facilitate suicide prevention, but it is unclear to what extent suicidal posts on SNS may induce contagion. Overall finding unclear.	NA.
Silenzio et al (2009) [56]<br>*n*=100,014	SNS, general.	Descriptive study, 16-24 years.	SNS may facilitate suicide prevention and addressing other health concerns. Overall finding positive.	NA
Takahashi et al (2009) [57]<br>*n*=105	SNS, clinical.	Observational cross sectional study, 21-57 years.	SNS can offer support but harms include worsening depressive symptoms. Overall finding mixed.	No.
Veretilo & Billick (2012) [59]<br>*n*=1	SNS, clinical.	Case study, 52 years.	Facebook use substantially reduced reclusiveness, manic symptoms and increased compliance with therapy and medication. Overall finding positive.	NA.
Wright et al (2013) [58]<br>*n*=361	SNS, general.	Survey, 18-51 years.	Interpersonal motives (eg "I use Facebook to talk to friends and people I know") predicted increased face-to-face and computer mediated communication competence, increased satisfaction with social support from face to face and Facebook interactions, and lower depression scores. Overall finding positive.	NA.

Note. OSG = online support groups, SNS = social networking sites.
